# Effects of social isolation on quality of life in elderly adults

**DOI:** 10.1371/journal.pone.0276590

**Published:** 2022-11-03

**Authors:** Roger D. Newman-Norlund, Sarah E. Newman-Norlund, Sara Sayers, Alexander C. McLain, Nicholas Riccardi, Julius Fridriksson

**Affiliations:** 1 Department of Communication Sciences, Arnold School of Public Health, University of South Carolina, Columbia, South Carolina, United States of America; 2 Department of Epidemiology and Biostatistics, Arnold School of Public Health, University of South Carolina, Columbia, South Carolina, United States of America; 3 Department of Psychology, College of Arts and Sciences, University of South Carolina, Columbia, South Carolina, United States of America; PLOS (Public Library of Science), UNITED KINGDOM

## Abstract

Prolonged periods of social isolation are known to have significant negative health consequences and reduce quality of life, an effect that is particularly pronounced in older populations. Despite the known deleterious effects of social isolation, a key component of the response to the COVID-19 pandemic has been the issuance of *stay at home* and/or *shelter in place* orders. Relatively little is known about the potential effects these periods of social isolation could have on older adults, and less still is known about potential risk factors or protective factors that modulate these effects. Here, we describe results from a longitudinal study in which we measured quality of life both prior to and immediately following a one-month period of social isolation associated with the issuance and revocation of a *shelter in place* order (April 6, 2020 through May 4, 2020) in the state of South Carolina. Healthy adult participants (N = 62) between the ages of 60 and 80 who had already completed quality of life questionnaires prior to isolation again completed the questionnaires following a one-month order to *shelter in place*. Quality of life significantly decreased during the social isolation period, with older participants showing the greatest declines. Participants with higher levels of physical activity and better physical/mental health going into the isolation period tended to show greater decreases in quality of life over time. These results highlight the negative consequences of even short bouts of social isolation for the elderly and suggest that reductions in social contact related to COVID-19 may have significant effects on mental health and emotional well-being, at least among older individuals.

## Introduction

The majority of research examining the negative effects of social isolation has targeted populations experiencing chronic social isolation (> 4 weeks), which is often coupled with other life-changing events. Rare conditions do exist in which humans are forced to endure temporary periods of social isolation (hospitalization, natural disasters, outbreaks of contagious disease, etc.). Referred to as situational social isolation [1], these periods of social isolation involve relative decreases in human-human interactions, social network size, frequency of contact with others, participation in social activities, and/or depth of social relationships [2]. The COVID-19 pandemic, which led governments around the world to impose varying levels of social distancing, presented a unique opportunity to study the effects of situational social isolation on quality of life in aging populations.

Chronic and acute social isolation have been shown to negatively impact quality of life [3–7]. While negative effects of social isolation manifest in persons of all ages [8], numerous studies indicate that consequences are particularly pronounced in elderly populations [2, 3, 9–17]. Although the negative impact of social isolation on elderly individuals is well-documented, less is known about the precise mechanisms underlying perceived changes in quality of life due to situational social isolation. Research indicates that the negative effects of social isolation on quality of life may be mediated by physical health [12, 18–23] as well as emotional well-being [12, 24–27]. In addition, there is evidence that the negative effects of social isolation may be modulated by socioeconomic status (SES), with lower SES individuals experiencing more severe adverse consequences [28–31].

The current study was designed to explore the potential impact of short-term (one month) situational social isolation in a convenience sample of elderly individuals (ages 60–80) who were impacted by South Carolina’s mandatory statewide *shelter in place* order, which went into effect on April 6, 2020 and was lifted on May 4, 2020. We predicted that self-reported quality of life would be significantly lower following the *shelter in place* order, and that changes in self-reported quality of life would vary as a function of chronological age, with older participants showing larger decreases in quality of life during social isolation than younger participants. Based on evidence suggesting a mediating role of mental and physical health on the effects of social isolation, we predicted that physical and mental health going into isolation would modulate the effects of social isolation. A better understanding of the effects of isolation resulting from COVID-19, both short-term and long-term, has potential to inform the implementation of both prescriptive and preventative health policy changes in the future.

## Methods

### Participants

A total of 62 (41 female, 21 male) participants who had previously completed testing for the Aging Brain Cohort (ABC) study [32], a multimodal study on healthy aging sponsored by the University of South Carolina (Columbia, SC), participated in the current study by repeating the remote-assessment portion of the study protocol administered prior to social isolation. Inclusion criteria included age between 60 and 80 at time of initial testing and ability to understand written and spoken English. Exclusion criteria included history of stroke, neurological disorder, and presence of any current chronic condition that would preclude participation in the study. The mean age of the sample was 67.35 (**SD** = 5.52) and the average Hollingshead SES score was 47.61 (**SD** = 12.55) [33]. This study was conducted in accordance with the Declaration of Helsinki. This study was approved by the University of South Carolina Institutional Review Board (IRB), and all subjects provided consent prior to study participation.

### Testing timeline

**Fig 1:**
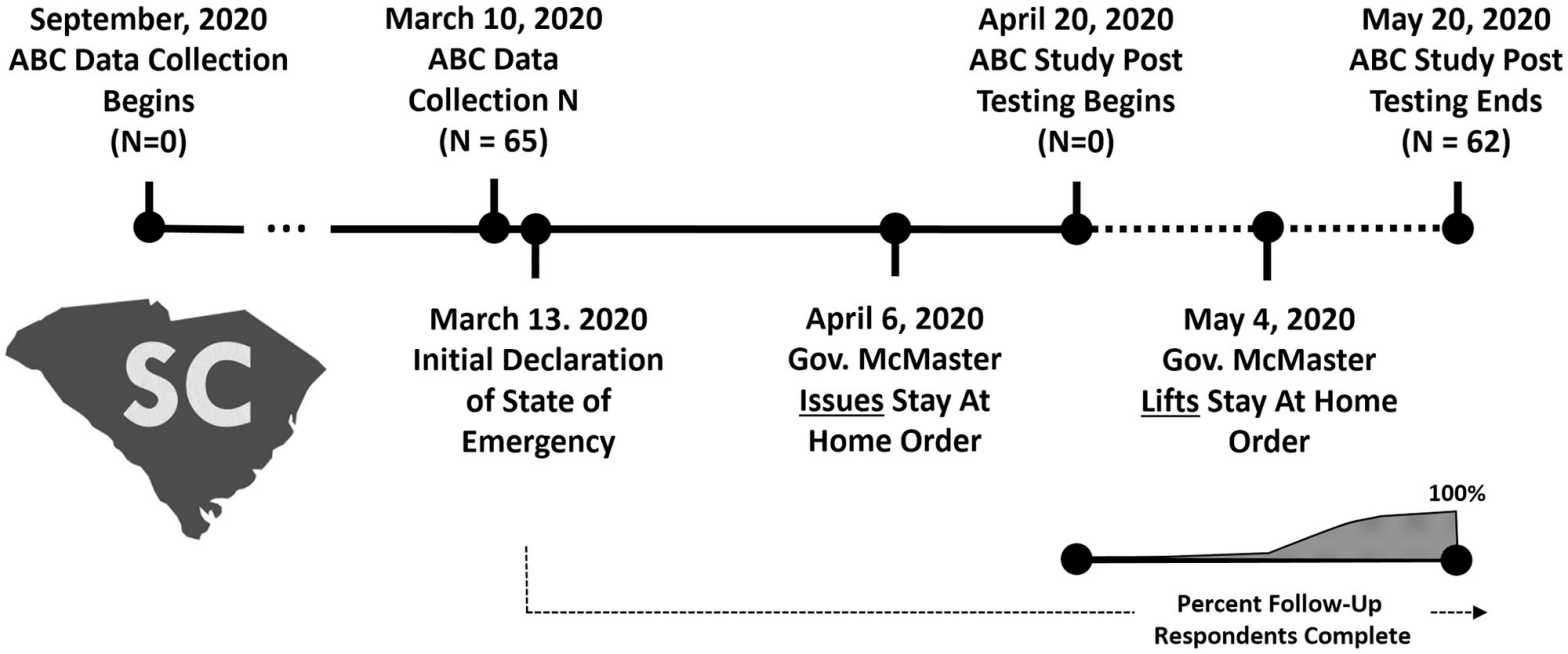
Timeline for recruitment and testing in the current study.

Data were collected from all participants at two different time points. Pre-isolation data were collected from participants between the dates of September 10, 2019 and April 6, 2020. Follow-up surveys were all completed once social isolation started between April 20 and May 20, 2020, with the majority (90%, N = 56) of participant surveys being completed between May 4 and May 10, 2020 (Fig 1).

### Measures

Questionnaires were administered using Research Electronic Data Capture (REDCap) hosted at the University of South Carolina [34] online, or by mail, if requested. In addition to providing basic demographic data, participants completed a number of qualitative and quantitative measures prior to and following social isolation. Quality of Life Inventory (QOLI^®^) was used to measure changes in overall perceived quality of life [35, 36], as well as changes in four factors of quality of life, achievement (QOLI_ACH_), self-expression (QOLI_SEP_), relationships (QOLI_REL_) and surroundings (QOLI_SUR_) identified in earlier research [37]. Total QOLI scores prior to isolation (QOLI_TOTPRE_) as well as QOLI scores post isolation (QOLI_TOTPOST_) were used to calculate a difference score (QOLI_TOTDIFF_) representing the change from pre to post assessment (i.e. QOLIPOST—QOLI_PRE_). Longitudinal changes in the four QOLI factors (QOLI_ACHDIFF_, QOLI_SEPDIFF_, QOLI_RELDIFF_ and QOLI_SURDIF_) were calculated in the same way (post—pre). Thus, QOLI_TOTDIFF_scores which were negative, represented longitudinal declines in QOLI, whereas QOLI_TOTDIFF_ difference scores which were positive represented longitudinal increases in QOLI. The extent to which participants were engaging in physical activity at both timepoints was assessed using the Physical Activity Scale for the Elderly (PASE) [38]. Physical and mental health, as represented by the physical and mental health summary scores [39], were assessed using the PROMIS^®^-29 Profile V20 [40]. For the PACE, physical and mental health scales, we used both pre-isolation scores (PACE_TOTPRE_, PROMISPH_TOTPRE_, PROMISMH_TOTPRE_) and difference scores (PACE_TOTDIFF_, PROMISPH_TOTDIFF_, PROMIS_TOTDIFF_, calculated as post-pre scores) in our analysis. Participants also filled out relevant PROMIS-29 subscales, including the social isolation, emotional support, ability to participate and instrumental support subscales. Finally, the 3-Item Loneliness Scale [41] provided a measure of feelings of loneliness, a construct distinct from–but related to–feelings of social isolation [42]. For all quantitative measures, except the loneliness scale, higher numbers were considered to be better. Correlations were computed using the R software Package for a Fast Calculation to Semi-partial Correlation Coefficients Communications for Statistical Application and Methods [43].

Participants also responded to open-ended, qualitative questions regarding the influence of social isolation on various components of their lives (i.e. “How much has COVID-19 related social isolation impacted the following domains of your life: work, family, medical, social/emotional, nutrition/exercise and access”).

## Results

Initially, we conducted statistical tests to confirm that the *shelter in place* order was associated with changes in feelings of social isolation and loneliness. As predicted, feelings of social isolation, as measured by the PROMIS-29 social isolation subscale, were significantly greater following the *shelter in place* order, t(55) = 2.07, p < 0.05, one-tailed, indicating that individuals felt more isolated following the order. Perceived loneliness, as measured by the 3-Item Loneliness Scale, did not significantly change, t(60) = -0.33, p > 0.05, one-tailed, between the two timepoints. Item-by-item, post hoc t-tests further exploring longitudinal changes in perceived loneliness separately for each of the three questions that comprise the test revealed that the extent to which participants felt like they were “lacking companionship” or “feeling left out” did not vary with feelings of isolation (p’s > 0.1). However, participants’ responses to the third question, which evaluated how “socially isolated” they were feeling, were significantly higher following isolation, t(60) = -2.21, p < 0.05, two-tailed. Participants reported a number of changes associated with social isolation including spending less time with family (37/62 = 60%), changes in work patterns (24/62 = 39%), changes in stress/worry/anxiety/depression/mood (27/62 = 44%), decreases in physical activity (22/62 = 35%), postponement/cancellation of health/dental care (15/62 = 24%), and changes in food/product availability (6/62 = 10%).

**Fig 2:**
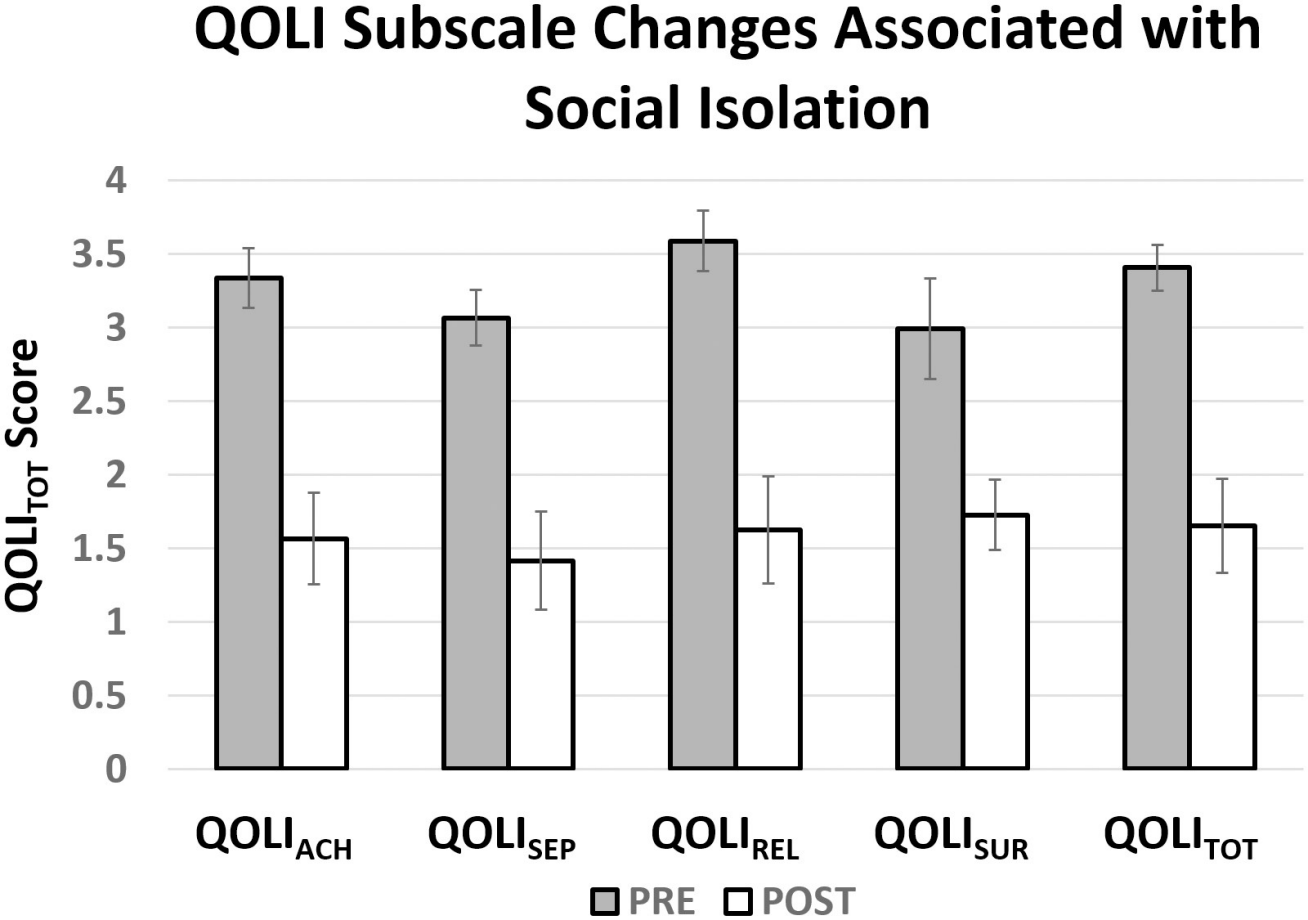
Social isolation related changes in quality of life. Pre- and post-isolation scores on four factors of the QOLI measure. These four factors were identified by O’Cleirigh and Safren [37] (N = 62). Ratings were significantly lower post-isolation for all four factors. **QOLI**_**ACH**_ = quality of life related to achievement; **QOLI**_**SEP**_ = quality of life related to self-expression, **QOLI**_**REL**_ = quality of life related to relationships; **QOLI**_**SUR**_ = quality of life related to surroundings; **QOLI**_**TOT**_ = quality of life total score.

Results from linear mixed models with random intercepts comparing quality of life scores prior to (QOLI_TOTPRE_, **M** = 3.40, **SD** = 1.19) and following social isolation (QOLI_TOTPOST_, **M** = 1.65, **SD** = 2.48) were significant (p < 0.001), indicating that perceived quality of life decreased from pre- to post-isolation testing. A series of four additional linear mixed models confirmed that isolation induced changes in self-reported quality of life, when calculated separately for items in the achievement (QOLI_ACHDIFF_), self-expression (QOLI_SEPDIFF_), relationships (QOLI_RELDIFF_), and surroundings (QOLI_SURDIFF_) factors, were all statistically significant (Fig 2) (Table 1), even after controlling for age, sex, and SES. During social isolation, 51/62 (84%) participants experienced decreases in quality of life (**M** = -2.27, **SD** = 2.86), whereas 11/62 (16%) of participants experienced increases in quality of life (**M** = 0.64, **SD** = 0.60).

**Table 1:** Comparison of QOLI measures before and after social isolation.

Measures	M(pre)	SD(pre)	M(post)	SD(post)
QOLI_TOT_**	3.4	1.19	1.65	2.48
QOL1_ACH_**	3.34	1.45	1.56	2.43
QOL1_SEP_**	3.06	1.35	1.41	2.63
QOL1_REL_**	3.59	1.43	1.62	2.87
QOL1_SUR_*	2.99	1.17	1.72	2.68

Note: Pre-isolation and post-isolation scores were significantly different for all measures, both adjusted and unadjusted (age, sex, SES). **QOLI**_ACH_ = quality of life related to achievement; **QOLI**_SEP_ = quality of life related to self-expression, **QOLI**_REL_ = quality of life related to relationships; **QOLI**_SUR_ = quality of life related to surroundings; **QOLI**_T0T_ = quality of life total score.

*p < 0.005,

**p < 0.001

**Fig 3:**
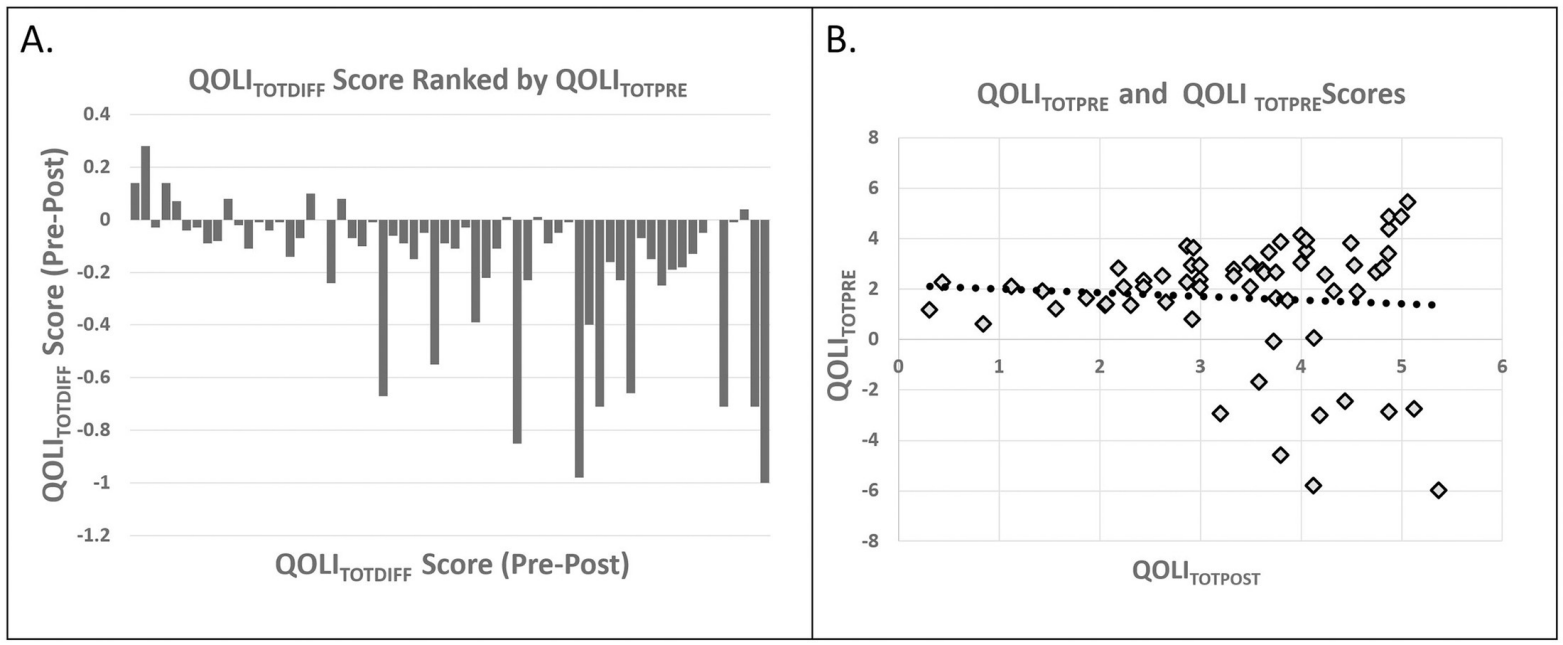
(**A**) Rank-order plot showing change in quality of life scores as they relate to quality of life ratings going into social isolation. Participants with higher quality of life scores at the beginning of social isolation (right side of graph) showed greater isolation-induced decreases in quality of life. **QOLI**_**TOTPRE**_ = pre-isolation quality of life scores; **QOLI**_**TOTDIFF**_ = post-isolation quality of life scores = pre-isolation quality of life scores. (**B**) Correlation between **QOLI**_**TOTPRE**_ and **QOLI**_**TOTPOST**_.

A Spearman rank correlation revealed that higher QOLI_TOTPRE_ scores were negatively associated with QOLI_TOTDIFF_ scores, r(62) = -0.51, p = 0.000018 (Fig 3), indicating that individuals with higher QOLI scores prior to social isolation showed greater decreases in QOLI across social isolation. This was also true after controlling for age and SES, r(62) = -0.49, p = 0.00074. The correlation between baseline (QOLI_TOTPRE_) and post isolation (QOLI_TOTPOST_) scores was not statistically significant (ns, p = 0.584 unadjusted, p = 0.711 adjusted) (Fig 3). Additionally, age was not significantly associated with higher initial QOLI_TOTPRE_ scores, r(62) = 0.12, p = 0.37.

**Fig 4:**
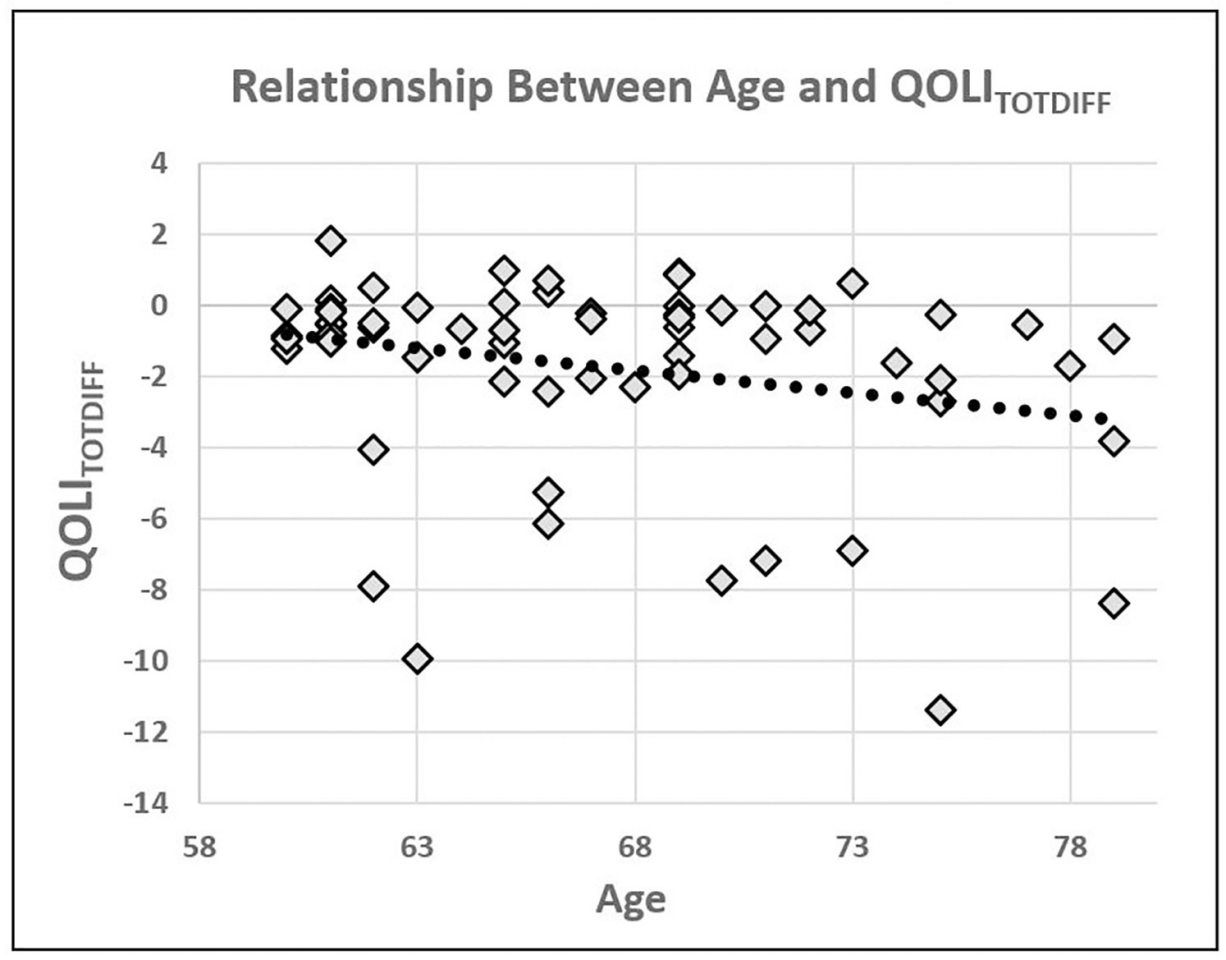
Plot showing the significant correlation between participant age and QOLI_TOTDIFF_. Older participants showed greater decreases in quality life as measured by the QOLI when comparing pre and post-isolation scores.

A Spearman rank correlation indicated that there was a significant negative correlation between age and QOLI_TOTDIF_, r(62) = 0.23, p = 0.04, one-tailed, suggesting that participants who were older, exhibited greater pre-post decreases in QOLI scores (Fig 4). When looking at each of the four QOLI factors separately, Spearman rank correlations revealed that there was a significant negative correlation between age and QOLI_ACHDIFF_, r(62) = 0.26, p = 0.04 as well as between age and QOLI_RELDIFF_, r(62) = 0.25, p = 0.049. There was, however, neither a significant correlation between age and QOLI_ACHSUR_, r(62) = 0.07, p = 0.575, nor a significant correlation between age and QOLI_SEPDIF_, r(62) = 0.162, p = 0.209.

**Fig 5:**
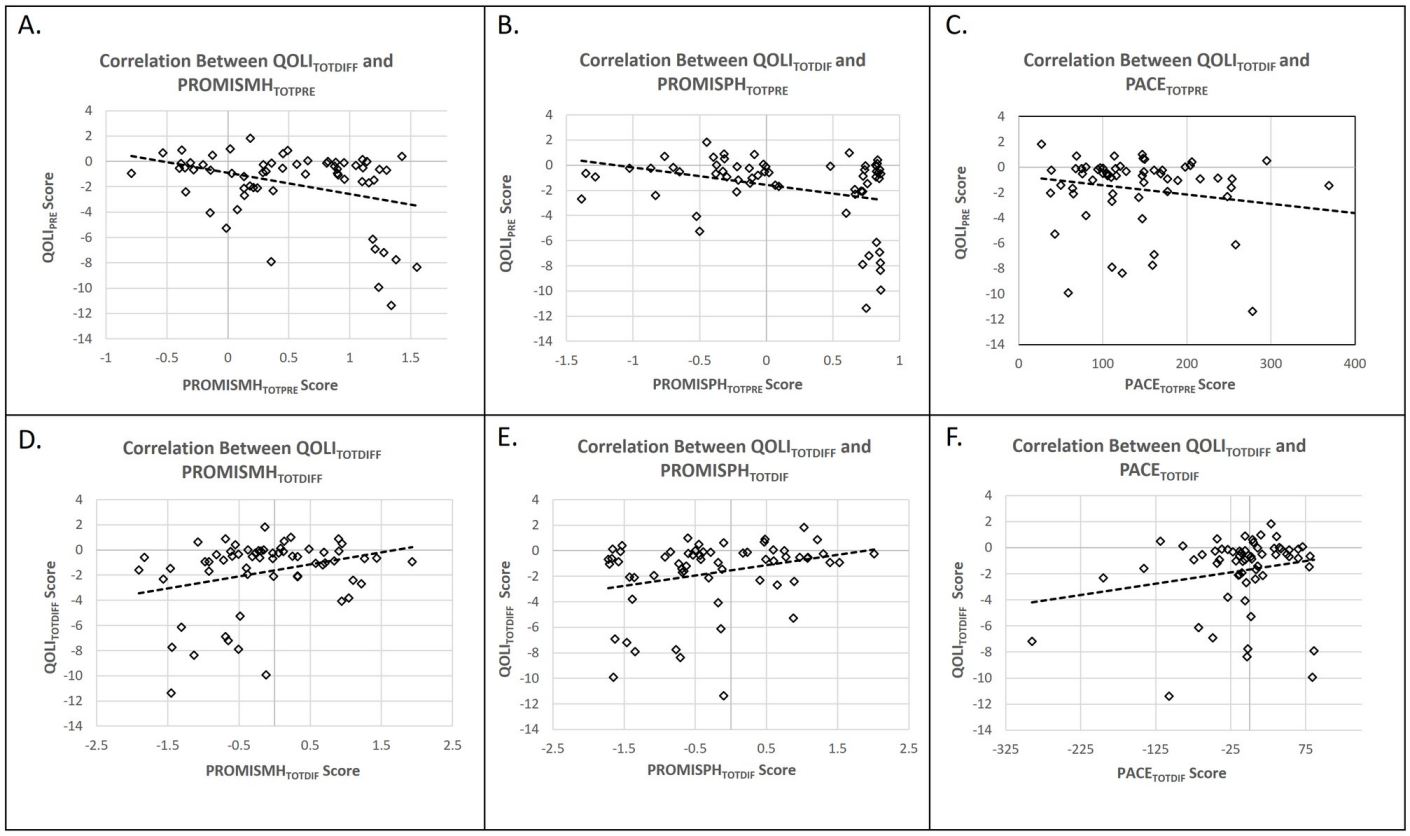
(**A-C**) Plots showing the relationship between *baseline* PROMIS mental/physical health summary scores and PACE scores and changes in QOLI. (**D-F**) Plots showing the relationship between *changes* in the PROMIS mental/physical health summary scores and PACE scores and changes in QOLI.

Spearman rank correlations were used to assess the association between the PACE PROMIS-29 physical and mental health scores measured at baseline (PACE_TOTPRE_, PROMISPH_TOTPRE_, PROMISMH_TOTPRE_) and QOLI_TOTDIFF_ scores. The association between the PROMISPH_TOTPRE_ and QOLI_TOTDIFF_ scores, was not significant without adjusting for age and SES, r(62) = -0.22, p = 0.09, but was significant after adjusting for age and SES, r(62) = -0.28, p = 0.035. Regarding PROMISMH_TOTPRE_, there was an insignificant, but trending correlation with QOLI_TOTDIFF_ score, both before, r(62) = -0.23, p = 0.075, and after controlling for age and SES, r(62) = -0.204, p = 0.124. A Spearman rank correlation indicated that PACE_TOTPRE_ was not significantly correlated with QOLI_TOTDIFF_ scores, r(62) = -0.179, p = 0.164. Changes in PROMISMH_TOTDIFF_, PROMISPH_TOTDIFF_, and PROMISMH_TOTDIFF_ were not significantly correlated with QOLI_TOTDIFF_ (p’s > 0.15) (Fig 5).

## Discussion

### Social isolation and quality of life

The primary purpose of this study was to examine the effects of COVID-19 related social isolation on perceived quality of life in elderly individuals. Our results indicate that the one-month period of social isolation associated with South Carolina’s April 6th, 2020, *shelter in place* order led to a highly significant decrease in overall quality of life in our convenience sample of elderly individuals. This effect was pervasive in that all subfactors of the quality of life scale appear to have been affected to a similar degree: participants reported significantly lower quality of life in domains related to achievement, self-expression, relationships, and their surroundings.

These results are consistent with prior literature showing that chronic social isolation has negative effects on health [3–7], and our findings add to the literature by demonstrating that even relatively brief periods of social isolation can have considerable consequences for the elderly. Situational or acute feelings of loneliness have been associated with greater risk of all-cause mortality [1, 44]. Recent cross-sectional studies of the COVID-19 pandemic around the world are finding that quality of life and mental health have been negatively affected [45, 46], however, our study is unique in that the same group of participants were tested before and then immediately after a government enforced *stay at home* order. This allows for stronger claims about the relationship between mandated social isolation and changes in perceived quality of life. Overall, our data indicate that this acute period of social isolation was associated with substantial decreases in perceived quality of life in the majority of older individuals included in our sample.

Notably, not all participants in the current study showed a decrease in perceived quality of life as a function of social isolation. In total, only 11 participants (18%) showed a numerical increase in perceived quality of life, and the average change score in increasers was quite small (0.6 points). A total of 51 participants (82%) experienced decreases in quality of life, and the size of this change was relatively large (-2.27 points). We did offer participants the opportunity to share their experiences with us via a series of open-ended questions. Participants reported less socialization with friends and family, changes in work schedules, changes in physical activity (mostly negative, but a few positive), postponement and cancellation of medical/dental procedures among the primary effects of the *shelter in place* order. Future qualitative studies, which might involve creation of focus groups to identify common themes, or conduct structured interviews of individuals during and/or after social isolation, would certainly be useful in expanding our understanding, not only of the relationship between social isolation and QOLI, but also the experiences that impact it the most.

### Social isolation and age/SES

Based on previous literature showing that older participants suffer greater negative consequences from social isolation [2, 3, 9–17], we predicted that older individuals would disproportionately suffer the ill-effects of social isolation. Our results support this hypothesis and are consistent with previous research. Older participants did demonstrate significantly greater decreases in quality of life than younger participants, and this effect was consistent across the four quality of life subcomponents (achievement, self-expression, surroundings, or relationships) [37]. One might argue that older participants generally started with greater QOLI scores and thus, had more space for negative change. However, the fact that there was no significant correlation between age and baseline QOLI scores argues against this claim. Interestingly, age was not associated with any of the other cross-sectional measures of interest in the study except loneliness (older people reported being more lonely) and instrumental support. The positive correlation between loneliness and age makes sense given the correlation between age and marital status (higher age → higher chance of living alone, r(62) = -0.34, p < 0.005). Instrumental support captures the extent to which an individual has someone to help them with meals, errands, chores, medical care, cleaning, etc. This role is typically filled by a spouse, so this too may be explained by the known inverse correlation between age and marital status. The fact that loneliness and instrumental support were, in and of themselves, not correlated with QOLI_TOTDIFF_, whereas age was, suggests that age, suggests that age encompasses a number of different variables, some of which may not have even been measured in this study. Although more research is certainly needed, our data are consistent with the idea that elderly individuals that are single or living alone are likely to be at greater risk for experiencing negative consequences due to even short periods of social isolation.

Interestingly, social isolation was not associated with changes in loneliness, as measured by the total score on the 3-Item Loneliness scale, but was associated with feelings of social isolation, as measured by the final question of the social isolation scale. This is important in light of recent evidence suggesting that loneliness, and not social isolation per se, may be the driving force behind some of the negative consequences of social isolation [42]. Evidence from the 2012 wave of the Health and Retirement Study suggests that the use of technology, particularly e-mail, social networking sites, video/phone calls, and instant messaging, can improve health in older adults [47]. While use of social technology may have kept feelings of loneliness in check, it is also important to note that digital literacy is associated with improved access to healthcare and health discussions [48–50]. Future studies should more directly examine the impact of using social media and tele-communication platforms on loneliness and quality of life in times of social isolation. Finally, it is possible that participants who resided with a significant other or family members may have this period of social isolation differently than those living alone, as the situation afforded them more opportunities to connect within other people. This is corroborated by the findings that many of the participants in the current study were married and living with their spouse/significant other (37/62 = 60%), and many participants indicated spending more time with their family/significant other after–as compared to before–the *shelter in place* order.

The current study measured SES using the Hollingshead scale, which is widely used in health disparities research [51]. Participants in this study reported an average SES of 47.6 (+- 12.55). To put this finding in context, the original Hollingshead paper divides SES into 5 strata (ranging from low to high: strata 1 < 20; strata 2 = 20–29; strata 3 = 30–39, strata 4 = 40–54, and strata 5 = 55–60) [33]. The highly significant effect of social isolation on quality of life is particularly interesting given the relatively high SES of the current sample. This is not what we would have predicted based on previous research indicating protective effects of higher SES on health [28–31]. Taken together with the non-significant correlation between SES and changes in quality of life reported here, our data suggest that higher SES status does not necessarily protect elderly individuals from the negative effects of social isolation, and that researchers should not simply assume that high SES protects against negative effects of COVID-19-induced social isolation. We would like to note that one limitation of the current study is that there were relatively few participants with very low SES. Clearly, this issue is one that could be addressed by future studies with more diverse populations (in terms of SES) and larger sample sizes.

### Influence of mental and physical health/fitness on the effects of social isolation

Regarding our measures of physical activity, physical health, and mental health, we expected those with higher initial scores to experience smaller reductions in quality of life during the *shelter in place* order, i.e. that these would act as protective factors [52, 53]. In general, our data are inconsistent with these predictions. While not all of our results were statistically significant, the trends we report were all in the same direction, indicating that better physical activity, physical health, and mental health may be risk factors as opposed to protective factors. While there are many potential explanations for this effect, one possibility is that physical activity, mental health, and physical health prior to isolation were supported by social interactions and that the removal of opportunities for social interaction led to the observed decreases in quality of life. Rather than representing a protective factor, higher measures of overall health (when dependent on social interaction) may actually identify individuals at high risk for potentially large swings in quality of life during periods of isolation. Although further research should be conducted to verify these effects in a larger and more diverse sample, our data suggest that health professionals interested in minimizing the negative effects of isolation on quality of life should consider policies directed at maintaining physical and mental health in specific groups.

Interestingly there was no significant relationship between changes in quality of life and changes in the physical activity or physical and mental health measures. This is a somewhat puzzling result given the significant relationship between these variables and initial quality of life scores. Separate, post hoc tests exploring the possibility that changes in a one specific factor or quality of life were related to these measures also produced negative results. The simplest explanation for this finding is that quality of life is too complex to be captured by concomitant changes in exercise, physical health, and mental health alone. Indeed, social isolation is certainly associated with changes in multiple other realms, including diet, leisure activities, technology usage, spousal interactions, exposure to unique experiences, etc., not to mention the possibility of interactions between all of these factors. Clearly, there isa need for more complex and comprehensive models to explain how changes in physical activity, physical health, and mental health are related to changes in quality of life during periods of social isolation.

## Study limitations

A number of limitations should be considered when interpreting findings from the current study. First and foremost, this study relied primarily on self-reported data which may be inaccurate, or incomplete, and can be influenced by factors like perceived social desirability and/or recall error. This limitation is typical of survey-based studies, especially those conducted remotely (using computers). Since our study intervention was not planned (i.e., COVID-19-based isolation was an unexpected event), we were only able to calculate pre- and post-isolation data for the subset of tests we administered to participants prior to the *shelter in place* order. Although we tried to capture some additional information using open-ended questions targeting the effects of social isolation across various domains, these are not a substitute for reliable and valid measures of related constructs that could be used in future, planned studies of the effects of social isolation. Additionally, we acknowledge that our measure of SES, the Hollingshead Four-Factor Index [33] is not without limitations. In particular, the occupational rankings do not take into account the current economic/employment makeup of South Carolina and may not accurately reflect earnings for specific types of jobs.

Critically, quality of life is a complex and subjective concept, based on myriad factors including but not limited to physical health, happiness, usefulness, wealth, and the quality of both personal and interpersonal relationships. While the current study was able to explore both overall quality of life, as well as various factors identified in previous research [37], more could be done to understand the complexity and nuance of this concept. For example, the study which identified the four factors examined in this paper was based on factor analysis of responses from a clinical population, namely HIV survivors. It would be useful to examine whether or not these, or similar, four factors emerge in other clinical populations (e.g., cancer, stroke), or even other non-clinical populations. As mentioned in the results section, responses to open-ended questions were revealing, and suggest the possibility of future avenues of study. Qualitative studies involving semi-structured interviews or group forum discussions could lead to an even deeper understanding of what people find to be important in evaluating their own quality of life. In all likelihood, these evaluations depend on other, as-yet-to-be-identified variables, including past experiences and future goals; additionally, other cultures likely experience social isolation differently, depending on the relative value of social contact, exercise, etc. in the perception of QOLI.

## Conclusions

The response to COVID-19 necessarily leads to increases in social isolation, which is known to have negative health consequences, especially for elderly individuals. Our results demonstrate that even a short period of social isolation (one month) can affect quality of life, that the magnitude of this effect varies as a function of age, and that, surprisingly, individuals with higher pre-isolation mental and physical health appear to suffer greater decreases in quality of life. These findings highlight the critical importance of understanding the relationship between COVID-19-related social isolation and may be useful to health professionals designing policy approaches to minimizing the negative consequences of social isolation.
